# On Nonlinear Bending Study of a Piezo-Flexomagnetic Nanobeam Based on an Analytical-Numerical Solution

**DOI:** 10.3390/nano10091762

**Published:** 2020-09-06

**Authors:** Mohammad Malikan, Victor A. Eremeyev

**Affiliations:** 1 Department of Mechanics of Materials and Structures, Faculty of Civil and Environmental Engineering, Gdansk University of Technology, 80-233 Gdansk, Poland; mohammad.malikan@pg.edu.pl; 2 Laboratory of Mechanics of Biomaterials, Research and Education Center “Materials”, Don State Technical University, Gagarina sq., 1, Rostov on Don 344000, Russia

**Keywords:** flexomagnetic, nanobeam, large deflection, NSGT, Galerkin method, Newton–Raphson method

## Abstract

Among various magneto-elastic phenomena, flexomagnetic (FM) coupling can be defined as a dependence between strain gradient and magnetic polarization and, contrariwise, elastic strain and magnetic field gradient. This feature is a higher-order one than piezomagnetic, which is the magnetic response to strain. At the nanoscale, where large strain gradients are expected, the FM effect is significant and could be even dominant. In this article, we develop a model of a simultaneously coupled piezomagnetic–flexomagnetic nanosized Euler–Bernoulli beam and solve the corresponding problems. In order to evaluate the FM on the nanoscale, the well-known nonlocal model of strain gradient (NSGT) is implemented, by which the nanosize beam can be transferred into a continuum framework. To access the equations of nonlinear bending, we use the variational formulation. Converting the nonlinear system of differential equations into algebraic ones makes the solution simpler. This is performed by the Galerkin weighted residual method (GWRM) for three conditions of ends, that is to say clamp, free, and pinned (simply supported). Then, the system of nonlinear algebraic equations is solved on the basis of the Newton–Raphson iteration technique (NRT) which brings about numerical values of nonlinear deflections. We discovered that the FM effect causes the reduction in deflections in the piezo-flexomagnetic nanobeam.

## 1. Introduction

To study the flexomagnetic (FM) effect and to better identify it, one can use the family close to it, that is, the piezomagnetic effect. In piezomagnetic, simply by compressing or stretching materials, an internal magnetic field is created in them. The piezomagnetic effect and its application can be seen in many materials and structures. However, in addition to these very useful applications, there is an important drawback that this effect can only exist in about 20 crystal structures with a specific symmetrical classification. However, there is no such limit to the FM effect, and materials with wider classes of symmetry can cause such a phenomenon. The flexomagnetic effect can be very strong and effective, so that it may one day be used in nanosensors or nanometer actuators. As a brief explanation of the FM effect, it can be noted that by bending an ionic crystal, the atomic layers are drawn inside it, and it is clear that the outermost layer will have the most tension. This difference in traction in different layers can cause ions to transfer to the crystal so much that they eventually create a magnetic field. In other words, bending some materials creates a magnetic field, a corresponding phenomenon called flexomagnetic effect. The effect of strain gradients shows that the importance of the FM effect in micro and nano systems is comparable to that of piezomagnetic and even beyond. Additionally, flexomagnetic, unlike piezomagnetic, can be found in a wider class of materials. This means that compared to piezomagnetic, which is invalid and inefficient in materials with central symmetry, there is an FM effect in all biological materials and systems. These traits have led to a growing interest in and research into the flexomagnetic effect in recent years [1,2]. Currently, the role of the flexomagnetic effect in the physics of dielectrics has been investigated in some studies and has shown promising practical applications [3,4,5,6,7]. On the other hand, the difference between theoretical and experimental results shows a limited understanding in this field. This study examines current knowledge of FM in engineering.

The flexomagnetic effect exists in many solid dielectrics, soft membranes, and biological filaments. The flexomagnetic effect is introduced as the effect of size-dependent electromagnetic coupling due to the presence of strain gradients and magnetic fields, and promises many applications in nano-electronic devices (with strong strain gradients). Just as the piezomagnetic effect is expected to have important applications in nano-engines and particles [8,9,10,11,12], so the FM effect can play this role as well. Different fields of science are used to study nanodielectrics by considering the FM effect. These significant parts can be examined from a chemistry and physics point of view, or they can be put under a magnifier in the engineering and industrial aspects. In the engineering aspects, the study of external factors on dielectrics and their mechanical and physical behavioral responses will naturally be the criterion for evaluation. The purpose of this study is to evaluate this aspect in static large deflection analysis of a nano actuator beam. A close look at the history of the study of the mechanical behavior of dielectrics by including the FM effect does not show many studies [13,14,15]. These studies have generally looked at small deformations (linear strains), which, while important, cannot be the criterion for designing dielectric nanobeams. Definitely, the deformations should be considered as large as possible to obtain a reasonable and reliable safety factor for optimizing these significant nano-electro-magneto-mechanical systems’ components.

The present work accounts for the large deflections by adding the nonlinear terms of Lagrangian strain using the von Kármán approach. The constitutive equations are expanded in line with the classical beam theory. It is worth mentioning that the small scale is fulfilled conforming to the second stress and strain gradients. These extra terms should result in two conflict responses, that is softening and hardening in the nanoscale structure based on the literature. We perform the solution of acquired equations, which govern the nonlinear bending of the nanobeam, on the basis of two step solution techniques. The first one is the Galerkin weighted residual method (GWRM) which converts the equations into nonlinear algebraic ones, then the Newton–Raphson technique (NRT), which solves the nonlinear system of algebraic equations and gives the numerical values of displacements into *x* and *z* directions. At last, pictorial results are evaluated to show the disagreements and dissimilarities betwixt linear deflection and nonlinear one for the piezo-flexomagnetic nanosize beam.

## 2. Mathematical Model

**Figure 1:**
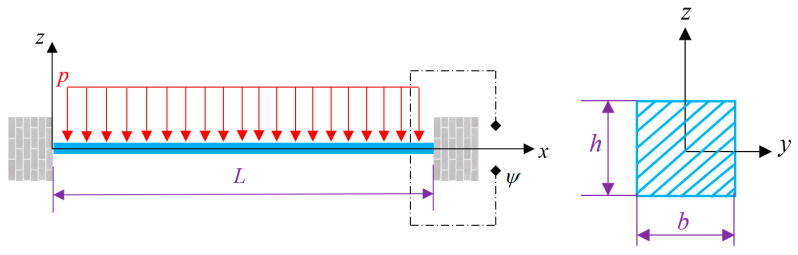
A square (*b* = *h*) PF-NB clamped at both ends and exposed to a lateral uniform static loading beside an external magnetic potential.

Let us consider a piezomagnetic-flexomagnetic nanobeam (PF-NB) with squared cross section of length and thickness *L* and *h*; see Figure 1. A uniform vertical static loading acts above the beam. A magnetic potential is joint to the beam to simulate and act as a magnetic field. Moreover, the *z*-axis is related to the transverse direction, whereas the neutral plane of the beam is coincident with the *x*-axis.

Follow up, the kinematic displacement for each node of the beam is utilized with the aid of the Euler–Bernoulli hypothesis [16,17]. Furthermore, the model is restricted with in-plane deformations. The rectangular displacements correspond with *u_1_* and *u_3_*, respectively, for axial and transverse directions. However, such displacements for neutral plane are, respectively, regarded with *u* and *w*. Thus, one can give accordingly

(1) $$u_{1}\left(x\;,z\right)=u\left(x\right)-z\frac{dw\left(x\right)}{dx}$$

(2) $$u_{3}\left(x,z\right)=w\left(x\right)$$

The Von Kármán assumption tells us that the nonlinear terms related to the *u* can be excluded from the Lagrangian strain formula because these terms are sufficiently small compared to the other terms [18,19,20,21,22,23,24]. The general Lagrangian strain can be mentioned as

(3) $$\varepsilon_{ij}=\frac{1}{2}\left(\frac{\partial u_{i}}{\partial x_{j}}+\frac{\partial u_{j}}{\partial x_{i}}+\frac{\partial u_{k}}{\partial x_{i}}\frac{\partial u_{k}}{\partial x_{j}}\right)$$

In regard to this approach, the nonzero nonlinear strain-displacement components can be derived as follows

(4) $$\varepsilon_{xx}=\frac{du}{dx}-z\frac{d^{2}w}{dx^{2}}+\frac{1}{2}{\left(\frac{dw}{dx}\right)}^{2}$$

(5) $$\eta_{xxz}=\frac{d\varepsilon_{xx}}{dz}=-\frac{d^{2}w}{dx^{2}}$$

where Equations (4) and (5) calculate, respectively, the longitudinal strain and its gradient.

The stress-strain magneto-mechanical coupling relations in the one-dimensional framework can be given owing to [13,14].

(6) $$\sigma_{xx}=C_{11}\varepsilon_{xx}-q_{31}H_{z}$$

(7) $$\xi_{xxz}=g_{31}\eta_{xxz}-f_{31}H_{z}$$

(8) $$B_{z}=a_{33}H_{z}+q_{31}\varepsilon_{xx}+f_{31}\eta_{xxz}$$

where $\sigma_{xx}$ is the static stress field component, $H_{z}$ is the magnetic field component, $B_{z}$ is the magnetic flux (induction) component, $C_{11}$ is the elastic modulus, $f_{31}$ is the component of the fourth-order flexomagnetic coefficients tensor, $a_{33}$ is the component of the second-order magnetic permeability tensor, $q_{31}$ is the component of the third-order piezomagnetic tensor, $g_{31}$ is the component of the sixth-order gradient elasticity tensor, and $\xi_{xxz}$ is the component of higher-order moment stress tensor.

The variational formulation accurately develops the characteristics relation of PF-NB, thusly

(9) δU−δW=0

where δ is the symbol of variation, U is the strain energies, and W is created works by outer objects. In such a way, the entire inner energy of the specimen is in the first variation which is equal to zero as well. The strain energy respecting magneto-mechanical composition can be variated just like this (the first variation)

(10) $$\delta U=\int_{V}^{}\left(\sigma_{xx}^{}\delta \varepsilon_{xx}^{}+\xi_{xxz}\delta \eta_{xxz}-B_{z}\delta H_{z}\right)dV$$

Equation (10) can be transformed with integration by parts on the basis of the one-dimensional displacement field previously assumed as follows

(11) $$\delta U=\delta \Pi_{U_{1}}^{Mech}+\delta \Pi_{U_{1}}^{Mag}+\delta \Pi_{U_{2}}^{Mech}+\delta \Pi_{U_{2}}^{Mag}$$

where

(12) $$\delta \Pi_{U_{1}}^{Mech}=-\int_{0}^{L}\left\{\frac{dN_{x}^{}}{dx}\delta u+\left[\frac{d^{2}M_{x}^{}}{dx^{2}}+\frac{d}{dx}\left(N_{x}^{}\frac{dw}{dx}\right)+\frac{d^{2}T_{xxz}}{dx^{2}}\right]\delta w\right\}dx$$

(13) $$\delta \Pi_{U_{1}}^{Mag}=-\int_{0}^{L}\int_{-h/2}^{h/2}\frac{dB_{z}}{dz}\delta \Psi dzdx$$

(14) $$\delta \Pi_{U_{2}}^{Mech}={\left\{N_{x}^{}\delta u-\left[M_{x}^{}+T_{xxz}\right]\frac{d\delta w}{dx}+\left[N_{x}^{}\frac{dw}{dx}+\frac{dM_{x}^{}}{dx}+\frac{dT_{xxz}}{dx}\right]\delta w\right\}|}_{0}^{L}$$

(15) $$\delta \Pi_{U_{2}}^{Mag}={\int_{0}^{L}\left(B_{z}\delta \Psi \right)|}_{-h/2}^{h/2}dx$$

where Ψ is the variable of magnetic potential. The resultants of the stress field can be introduced along the following lines

(16) $$N_{x}^{}=\int_{-h/2}^{h/2}\sigma_{xx}dz$$

(17) $$M_{x}^{}=\int_{-h/2}^{h/2}\sigma_{xx}zdz$$

(18) $$T_{xxz}=\int_{-h/2}^{h/2}\xi_{xxz}dz$$

In addition, the magnetic potential was introduced through the relation

(19) $$\frac{d\Psi }{dz}=-H_{z}$$

External forces (axial force as a result of the longitudinal magnetic field and the lateral loading) create work thermodynamically in the particles so that the mathematical relation in the first variation becomes [25].

(20) $$\delta W=\int_{0}^{L}\left[N_{x}^{0}\left(\frac{d\delta w}{dx}\frac{dw}{dx}\right)+p\left(x\right)\delta w\right]dx$$

in which $N_{x}^{0}$ is the in-plane longitudinal axial force, and p is the lateral load per unit length. Taking into account the closed circuit in conjunction with the inverse piezo case, the electrical boundary conditions can be attributed as below

(21) $$\Psi \left(+\frac{h}{2}\right)=\psi$$

(22) $$\Psi \left(-\frac{h}{2}\right)=0$$

in which ψ is the external magnetic potential on the upper surface. Making in hand Equations (8), (13), (15), (21) and (22) practicably expresses the magnetic field component and thereupon the magnetic potential function in line with thickness as follows [13,14]

(23) $$\Psi =-\frac{q_{31}}{2a_{33}}\left(z^{2}-\frac{h^{2}}{4}\right)\frac{d^{2}w}{dx^{2}}+\frac{\psi }{h}\left(z+\frac{h}{2}\right)$$

(24) $$H_{z}=z\frac{q_{31}}{a_{33}}\frac{d^{2}w}{dx^{2}}-\frac{\psi }{h}$$

On the basis of Equations (23) and (24), Equations (6)–(8) can be developed as

(25) $$\sigma_{xx}=C_{11}\left[\frac{du}{dx}+\frac{1}{2}{\left(\frac{dw}{dx}\right)}^{2}\right]-z\left(C_{11}+\frac{q_{31}^{2}}{a_{33}}\right)\frac{d^{2}w}{dx^{2}}+\frac{q_{31}^{}\psi }{h}$$

(26) $$\xi_{xxz}=-\left(g_{31}+\frac{q_{31}f_{31}z}{a_{33}}\right)\frac{d^{2}w}{dx^{2}}+\frac{f_{31}\psi }{h}$$

(27) $$B_{z}=q_{31}\left[\frac{du}{dx}+\frac{1}{2}{\left(\frac{dw}{dx}\right)}^{2}\right]-f_{31}\frac{d^{2}w}{dx^{2}}-\frac{a_{33}\psi }{h}$$

Subsequently, Equations (16)–(18) can be rewritten in detail as

(28) $$N_{x}^{}=C_{11}A\left[\frac{du}{dx}+\frac{1}{2}{\left(\frac{dw}{dx}\right)}^{2}\right]+q_{31}\psi$$

(29) $$M_{x}^{}=-I_{z}\left(C_{11}+\frac{q_{31}^{2}}{a_{33}}\right)\frac{d^{2}w}{dx^{2}}$$

(30) $$T_{xxz}=-g_{31}h\frac{d^{2}w}{dx^{2}}+f_{31}\psi$$

in which $N_{x}^{},\;M_{x}^{},\;T_{xxz}$ show the axial, moment, and hyper stress resultants, and $I_{z}^{}=\int_{A}z^{2}dA$ is the area moment of inertia.

The resultant magnetic axial stress, which is achieved due to the longitudinal magnetic field, based on Equation (28) can be determined as

(31) $$N_{}^{Mag}=q_{31}\psi$$

This force is supposed to act at both ends of the beam, thus

(32) $$N_{x}^{0}=N^{Mag}$$

Eventually, imposing Equation (9), one can write the governing equations in a combination of mechanical and magnetic conditions as

(33) $$\frac{dN_{x}^{}}{dx}=0$$

(34) $$\frac{d^{2}M_{x}^{}}{dx^{2}}+\frac{d^{2}T_{xxz}}{dx^{2}}+\left(N_{x}^{0}+N_{x}^{}\right)\frac{d^{2}w}{dx^{2}}+\frac{dN_{x}^{}}{dx}\frac{dw}{dx}-p=0$$

Due to being the nanobeam a size-dependent particle, the scale-dependent property should be substituted in Equations (33) and (34). In [26], the second strain gradient of Mindlin merged successfully with the nonlocal theory of Eringen. This model (NSGT) was incorporated in a lot of research performed on the nanoparticles in recent years—see e.g., [27,28,29,30,31,32,33,34,35,36,37,38] and many others—and can be a proper item at the nanoscale.

The model proposed by [26] can be compatible in our case as

$$\left(1-\mu \frac{d^{2}}{dx^{2}}\right)\sigma_{xx}^{NonLocal}=\left(1-l^{2}\frac{d^{2}}{dx^{2}}\right)\sigma_{xx}^{Local}$$

or as

(35) $$\left(1-\mu \frac{d^{2}}{dx^{2}}\right)\sigma_{xx}^{NonLocal}=\left(1-l^{2}\frac{d^{2}}{dx^{2}}\right)\left\{C_{11}\left[\frac{du}{dx}+\frac{1}{2}{\left(\frac{dw}{dx}\right)}^{2}\right]-z\left(C_{11}+\frac{q_{31}^{2}}{a_{33}}\right)\frac{d^{2}w}{dx^{2}}+\frac{q_{31}^{}\psi }{h}\right\}$$

in which $\mu \left(nm^{2}\right)$ is the nonlocal parameter, and l(nm) is the strain gradient parameter. Thus, l>0 establishes a nonzero strain gradient into the model, and $\mu ={\left(e_{0}a\right)}^{2}$ is the parameter defining nonlocality. It is germane to note that both scale parameters are dependent on the physics of the model and cannot be material constants [39,40]. This means the parameters are not constant values, something like an elasticity modulus for each material.

To implement the influence of size effects into the equations, Equation (35) is plugged to Equations (28)–(30) as

(36) $$N_{x}-\mu \frac{d^{2}N_{x}}{dx^{2}}=\left(1-l_{}^{2}\frac{d^{2}}{dx^{2}}\right)\left\{C_{11}A\left[\frac{du}{dx}+\frac{1}{2}{\left(\frac{dw}{dx}\right)}^{2}\right]\right\}$$

(37) $$M_{x}-\mu \frac{d^{2}M_{x}}{dx^{2}}=\left(1-l_{}^{2}\frac{d^{2}}{dx^{2}}\right)\left\{-I_{z}\left(C_{11}+\frac{q_{31}^{2}}{a_{33}}\right)\frac{d^{2}w}{dx^{2}}\right\}$$

(38) $$T_{xxz}-\mu \frac{d^{2}T_{xxz}}{dx^{2}}=\left(1-l_{}^{2}\frac{d^{2}}{dx^{2}}\right)\left\{-g_{31}h\frac{d^{2}w}{dx^{2}}+f_{31}\psi \right\}$$

Equations (33) and (34) by means of Equations (36)–(38) can be derived in the framework of displacements, respectively, as series of models.

1.1. Piezo-flexomagnetic nanobeam (PF-NB)—Nonlinear case:

(39) $$C_{11}A\left[\frac{d^{2}u}{dx^{2}}+\frac{d^{2}w}{dx^{2}}\frac{dw}{dx}-l_{}^{2}\left(\frac{d^{4}u}{dx^{4}}+\frac{d^{4}w}{dx^{4}}\frac{dw}{dx}+3\frac{d^{3}w}{dx^{3}}\frac{d^{2}w}{dx^{2}}\right)\right]=0$$

(40) $$\begin{array}{l} -g_{31}h\frac{d^{4}w}{dx^{4}}+q_{31}\psi \frac{d^{2}w}{dx^{2}}-p-\mu \left(-g_{31}h\frac{d^{6}w}{dx^{6}}+q_{31}\psi \frac{d^{4}w}{dx^{4}}-\frac{d^{2}p}{dx^{2}}\right)\\ -\mu C_{11}A\left[\frac{du}{dx}+\frac{1}{2}{\left(\frac{dw}{dx}\right)}^{2}\right]\frac{d^{4}w}{dx^{4}}+C_{11}A\mu l_{}^{2}\left[\frac{d^{3}u}{dx^{3}}+\frac{d^{3}w}{dx^{3}}\frac{dw}{dx}+{\left(\frac{d^{2}w}{dx^{2}}\right)}^{2}\right]\frac{d^{4}w}{dx^{4}}\\ -\mu C_{11}A\left(\frac{d^{2}u}{dx^{2}}+\frac{dw}{dx}\frac{d^{2}w}{dx^{2}}\right)\frac{d^{3}w}{dx^{3}}+C_{11}A\mu l_{}^{2}\left(\frac{d^{4}u}{dx^{4}}+3\frac{d^{3}w}{dx^{3}}\frac{d^{2}w}{dx^{2}}+\frac{dw}{dx}\frac{d^{4}w}{dx^{4}}\right)\frac{d^{3}w}{dx^{3}}\\ -\mu C_{11}A\left(\frac{d^{4}u}{dx^{4}}+\frac{dw}{dx}\frac{d^{4}w}{dx^{4}}+3\frac{d^{3}w}{dx^{3}}\frac{d^{2}w}{dx^{2}}\right)\frac{dw}{dx}+C_{11}A\left[\frac{du}{dx}+\frac{1}{2}{\left(\frac{dw}{dx}\right)}^{2}\right]\frac{d^{2}w}{dx^{2}}\\ +C_{11}A\mu l_{}^{2}\left(\frac{d^{6}u}{dx^{6}}+\frac{dw}{dx}\frac{d^{6}w}{dx^{6}}+5\frac{d^{5}w}{dx^{5}}\frac{d^{2}w}{dx^{2}}+10\frac{d^{4}w}{dx^{4}}\frac{d^{3}w}{dx^{3}}\right)\frac{dw}{dx}\\ -C_{11}Al_{}^{2}\left[\frac{d^{3}u}{dx^{3}}+\frac{d^{3}w}{dx^{3}}\frac{dw}{dx}+{\left(\frac{d^{2}w}{dx^{2}}\right)}^{2}\right]\frac{d^{2}w}{dx^{2}}+C_{11}A\left(\frac{d^{2}u}{dx^{2}}+\frac{dw}{dx}\frac{d^{2}w}{dx^{2}}\right)\frac{dw}{dx}\\ -I_{z}\left(C_{11}+\frac{q_{31}^{2}}{a_{33}}\right)\left(\frac{d^{4}w}{dx^{4}}-l_{}^{2}\frac{d^{6}w}{dx^{6}}\right)-C_{11}Al_{}^{2}\left(\frac{d^{4}u}{dx^{4}}+3\frac{d^{3}w}{dx^{3}}\frac{d^{2}w}{dx^{2}}+\frac{dw}{dx}\frac{d^{4}w}{dx^{4}}\right)\frac{dw}{dx}=0 \end{array}$$

1.2. Piezo-flexomagnetic nanobeam (PF-NB)—Linear case:

(41) $$\begin{array}{l} -g_{31}h\frac{d^{4}w}{dx^{4}}+q_{31}\psi \frac{d^{2}w}{dx^{2}}-p-\mu \left(-g_{31}h\frac{d^{6}w}{dx^{6}}+q_{31}\psi \frac{d^{4}w}{dx^{4}}-\frac{d^{2}p}{dx^{2}}\right)\\ -I_{z}\left(C_{11}+\frac{q_{31}^{2}}{a_{33}}\right)\left(\frac{d^{4}w}{dx^{4}}-l_{}^{2}\frac{d^{6}w}{dx^{6}}\right)=0 \end{array}$$

2.1. Piezomagnetic nanobeam (P-NB)—Nonlinear case:

(42) $$C_{11}A\left[\frac{d^{2}u}{dx^{2}}+\frac{d^{2}w}{dx^{2}}\frac{dw}{dx}-l_{}^{2}\left(\frac{d^{4}u}{dx^{4}}+\frac{d^{4}w}{dx^{4}}\frac{dw}{dx}+3\frac{d^{3}w}{dx^{3}}\frac{d^{2}w}{dx^{2}}\right)\right]=0$$

(43) $$\begin{array}{l} q_{31}\psi \frac{d^{2}w}{dx^{2}}-p-\mu \left(q_{31}\psi \frac{d^{4}w}{dx^{4}}-\frac{d^{2}p}{dx^{2}}\right)-\mu C_{11}A\left[\frac{du}{dx}+\frac{1}{2}{\left(\frac{dw}{dx}\right)}^{2}\right]\frac{d^{4}w}{dx^{4}}\\ +C_{11}A\mu l_{}^{2}\left[\frac{d^{3}u}{dx^{3}}+\frac{d^{3}w}{dx^{3}}\frac{dw}{dx}+{\left(\frac{d^{2}w}{dx^{2}}\right)}^{2}\right]\frac{d^{4}w}{dx^{4}}\\ -\mu C_{11}A\left(\frac{d^{2}u}{dx^{2}}+\frac{dw}{dx}\frac{d^{2}w}{dx^{2}}\right)\frac{d^{3}w}{dx^{3}}+C_{11}A\mu l_{}^{2}\left(\frac{d^{4}u}{dx^{4}}+3\frac{d^{3}w}{dx^{3}}\frac{d^{2}w}{dx^{2}}+\frac{dw}{dx}\frac{d^{4}w}{dx^{4}}\right)\frac{d^{3}w}{dx^{3}}\\ -\mu C_{11}A\left(\frac{d^{4}u}{dx^{4}}+\frac{dw}{dx}\frac{d^{4}w}{dx^{4}}+3\frac{d^{3}w}{dx^{3}}\frac{d^{2}w}{dx^{2}}\right)\frac{dw}{dx}+C_{11}A\left[\frac{du}{dx}+\frac{1}{2}{\left(\frac{dw}{dx}\right)}^{2}\right]\frac{d^{2}w}{dx^{2}}\\ +C_{11}A\mu l_{}^{2}\left(\frac{d^{6}u}{dx^{6}}+\frac{dw}{dx}\frac{d^{6}w}{dx^{6}}+5\frac{d^{5}w}{dx^{5}}\frac{d^{2}w}{dx^{2}}+10\frac{d^{4}w}{dx^{4}}\frac{d^{3}w}{dx^{3}}\right)\frac{dw}{dx}\\ -C_{11}Al_{}^{2}\left[\frac{d^{3}u}{dx^{3}}+\frac{d^{3}w}{dx^{3}}\frac{dw}{dx}+{\left(\frac{d^{2}w}{dx^{2}}\right)}^{2}\right]\frac{d^{2}w}{dx^{2}}+C_{11}A\left(\frac{d^{2}u}{dx^{2}}+\frac{dw}{dx}\frac{d^{2}w}{dx^{2}}\right)\frac{dw}{dx}\\ -I_{z}\left(C_{11}+\frac{q_{31}^{2}}{a_{33}}\right)\left(\frac{d^{4}w}{dx^{4}}-l_{}^{2}\frac{d^{6}w}{dx^{6}}\right)-C_{11}Al_{}^{2}\left(\frac{d^{4}u}{dx^{4}}+3\frac{d^{3}w}{dx^{3}}\frac{d^{2}w}{dx^{2}}+\frac{dw}{dx}\frac{d^{4}w}{dx^{4}}\right)\frac{dw}{dx}=0 \end{array}$$

2.2. Piezomagnetic nanobeam (P-NB)—Linear case:

(44) $$q_{31}\psi \frac{d^{2}w}{dx^{2}}-p-\mu \left(q_{31}\psi \frac{d^{4}w}{dx^{4}}-\frac{d^{2}p}{dx^{2}}\right)-I_{z}\left(C_{11}+\frac{q_{31}^{2}}{a_{33}}\right)\left(\frac{d^{4}w}{dx^{4}}-l_{}^{2}\frac{d^{6}w}{dx^{6}}\right)=0$$

3.1. Nanobeam (NB)—Nonlinear case:

(45) $$C_{11}A\left[\frac{d^{2}u}{dx^{2}}+\frac{d^{2}w}{dx^{2}}\frac{dw}{dx}-l_{}^{2}\left(\frac{d^{4}u}{dx^{4}}+\frac{d^{4}w}{dx^{4}}\frac{dw}{dx}+3\frac{d^{3}w}{dx^{3}}\frac{d^{2}w}{dx^{2}}\right)\right]=0$$

(46) $$\begin{array}{l} -p+\mu \frac{d^{2}p}{dx^{2}}-\mu C_{11}A\left[\frac{du}{dx}+\frac{1}{2}{\left(\frac{dw}{dx}\right)}^{2}\right]\frac{d^{4}w}{dx^{4}}\\ +C_{11}A\mu l_{}^{2}\left[\frac{d^{3}u}{dx^{3}}+\frac{d^{3}w}{dx^{3}}\frac{dw}{dx}+{\left(\frac{d^{2}w}{dx^{2}}\right)}^{2}\right]\frac{d^{4}w}{dx^{4}}\\ -\mu C_{11}A\left(\frac{d^{2}u}{dx^{2}}+\frac{dw}{dx}\frac{d^{2}w}{dx^{2}}\right)\frac{d^{3}w}{dx^{3}}\\ +C_{11}A\mu l_{}^{2}\left(\frac{d^{4}u}{dx^{4}}+3\frac{d^{3}w}{dx^{3}}\frac{d^{2}w}{dx^{2}}+\frac{dw}{dx}\frac{d^{4}w}{dx^{4}}\right)\frac{d^{3}w}{dx^{3}}\\ -\mu C_{11}A\left(\frac{d^{4}u}{dx^{4}}+\frac{dw}{dx}\frac{d^{4}w}{dx^{4}}+3\frac{d^{3}w}{dx^{3}}\frac{d^{2}w}{dx^{2}}\right)\frac{dw}{dx}+C_{11}A\left[\frac{du}{dx}+\frac{1}{2}{\left(\frac{dw}{dx}\right)}^{2}\right]\frac{d^{2}w}{dx^{2}}\\ +C_{11}A\mu l_{}^{2}\left(\frac{d^{6}u}{dx^{6}}+\frac{dw}{dx}\frac{d^{6}w}{dx^{6}}+5\frac{d^{5}w}{dx^{5}}\frac{d^{2}w}{dx^{2}}+10\frac{d^{4}w}{dx^{4}}\frac{d^{3}w}{dx^{3}}\right)\frac{dw}{dx}\\ -C_{11}Al_{}^{2}\left[\frac{d^{3}u}{dx^{3}}+\frac{d^{3}w}{dx^{3}}\frac{dw}{dx}+{\left(\frac{d^{2}w}{dx^{2}}\right)}^{2}\right]\frac{d^{2}w}{dx^{2}}+C_{11}A\left(\frac{d^{2}u}{dx^{2}}+\frac{dw}{dx}\frac{d^{2}w}{dx^{2}}\right)\frac{dw}{dx}\\ -I_{z}C_{11}\left(\frac{d^{4}w}{dx^{4}}-l_{}^{2}\frac{d^{6}w}{dx^{6}}\right)-C_{11}Al_{}^{2}\left(\frac{d^{4}u}{dx^{4}}+3\frac{d^{3}w}{dx^{3}}\frac{d^{2}w}{dx^{2}}+\frac{dw}{dx}\frac{d^{4}w}{dx^{4}}\right)\frac{dw}{dx}=0 \end{array}$$

3.2. Nanobeam (NB)—Linear case:

(47) $$-p+\mu \frac{d^{2}p}{dx^{2}}-C_{11}I_{z}\left(\frac{d^{4}w}{dx^{4}}-l_{}^{2}\frac{d^{6}w}{dx^{6}}\right)=0$$

4.1. Classic beam—Nonlinear case:

(48) $$C_{11}A\left(\frac{d^{2}u}{dx^{2}}+\frac{d^{2}w}{dx^{2}}\frac{dw}{dx}\right)=0$$

(49) $$-p+C_{11}A\left[\frac{du}{dx}+\frac{1}{2}{\left(\frac{dw}{dx}\right)}^{2}\right]\frac{d^{2}w}{dx^{2}}+C_{11}A\left(\frac{d^{2}u}{dx^{2}}+\frac{dw}{dx}\frac{d^{2}w}{dx^{2}}\right)\frac{dw}{dx}-C_{11}I_{z}\frac{d^{4}w}{dx^{4}}=0$$

4.2. Classic beam—Linear case:

(50) $$-C_{11}I_{z}\frac{d^{4}w}{dx^{4}}=p$$

In what follows, we consider these cases in more details.

## 3. Solution Approach

The solution process here has two steps. The first step comes with the Galerkin weighted residual method (GWRM) on the basis of the admissible shape functions which satisfy boundary conditions. The second step is imposing the Newton–Raphson technique (NRT) in order to solve the system of nonlinear algebraic equations originated from GWRM. The following displacements were employed [41].

(51) $$u\left(x\right)=\sum_{m=1}^{\infty }U_{m}\frac{dX_{m}\left(x\right)}{dx}$$

(52) $$w\left(x\right)=\sum_{m=1}^{\infty }W_{m}X_{m}\left(x\right)$$

where $U_{m}$ and $W_{m}$ are unknown variables that determine displacements through two axes and should be computed, whereas $X_{m}\left(x\right)$ are shape functions, *m* is the axial half-wave number, and becomes m=1,2,…∞. The allowable shape functions given below satisfy end conditions as [41].

(53) $$S-S:\;X_{m}\left(x\right)=\sin\left(\frac{m\pi }{L}x\right)$$

(54) $$C-C:\;X_{m}\left(x\right)=\sin^{2}\left(\frac{m\pi }{L}x\right)$$

(55) $$C-F:\;X_{m}\left(x\right)=\sin\left(\frac{m\pi }{4L}x\right)\;\cos\left(\frac{m\pi }{4L}x\right)$$

in which S, C, and F mark one by one the simply-supported, clamped, and free end conditions. Here, e.g., C-F means a side of the beam is inserted in a clamping fixture and the opposite side is free and hanging.

Based on the Fourier sine series, the transverse load can uniformly behave on the nanobeam as the following form [42,43].

(56) $$p\left(x\right)=\sum_{m=1}^{\infty }\frac{4p_{0}}{m\pi }\sin\left(\frac{m\pi }{L}x\right)$$

in which $p_{0}$ is density of the lateral load. Inserting Equations (51), (52), and (56) into Equations (39)–(50), and integrating over the axial domain based on the GWRM approach, one can obtain

(57) $$\int_{0}^{L}\left[\eta \left(x\right)Y_{m}\right]dx=0$$

(58) $$\int_{0}^{L}\left[\xi \left(x\right)Z_{m}\right]dx=0$$

in which η and ξ are the first and second equations, respectively, and $Y_{m}$ and $Z_{m}$ show the residuals. Then, with ordering and arranging the aforesaid equations, one can receive the nonlinear algebraic system of two equations and two unknown variables (when considering *m* = 1). To solve such a system, there are several methods. As long as the NRT converged the results very quickly and accurately, this technique was employed here. A primary guess ($U_{0}$ and $W_{0}$) was required for results in this approach. We can express the first iteration as [44].

(59) $$U_{1}=U_{0}-J^{-1}\times A_{0}$$

(60) $$W_{1}=W_{0}-J^{-1}\times A_{0}$$

where *J* denotes the Jacobian matrix 2 × 2 and *A* is a vector 2 × 1.

(61) $$J=\frac{\partial A_{0}}{\partial x},$$

(62) $$A_{0}=e\left(\begin{array}{c} U_{0}\\ W_{0} \end{array}\right)$$

where *e* is the governing equations with placing the first guesses. As a matter of fact, Equations (59) and (60) are iterative equations that are

(63) $$U_{n+1}^{}=U_{n+1}^{}-J^{-1}\times A_{n+1}^{},$$

(64) $$W_{n+1}^{}=W_{n+1}^{}-J^{-1}\times A_{n+1}^{}$$

where *n* is the number of iterations to receive the convergence. A few iterations are enough to obtain the desired accuracy. It is worth mentioning that the convergence and the expected accuracy were completely dependent on the value of the primary guesses. Consequently, the solution led to numerical values of displacements along axial and transverse axes. To plot the results for large deflections, we needed to obtain the vertical displacement only, and the other will not be drawn.

## 4. Numerical Results and Discussion

### 4.1. Results’ Validity

Based on performing some comparative studies, the credit of the present results can be checked. In so doing, in Table 1 a pinned–pinned nanobeam under a distributed uniform force is compared with the linear schema. The maximum deflection which occurred at the center of the beam was in a nondimensional state as proposed by [21,45]. A good harmony among the deflections’ values is obviously seen from the Table. It is noteworthy that the classical dimensionless deflection is indicated by *e_0_a*/*L* = 0. From the Table, it is found that the nondimensional maximum deflection increased as the value of the nonlocal parameter increased.

For an explicit understanding, another comparison is tabulated by Table 2, for which a typical macroscale beam was utilized under both fixed ends. The present results are validated with those of the finite element method (FEM). Both the current and FEM approach are on the basis of linear analysis. As FEM benefits from shear deformations, it gives higher deflections. It is notable in the Table that enlarging the volume of the load resulted in the discrepancy of deflections. The FEM outcomes can be changeable due to many conditions in its process such as the number of elements, the kind of element, the number of nodes, and the algorithm of meshing, etc.

### 4.2. Discussion of the Problem

Here, just employing *n* = 4 gave the convergence in numerical results of the Newton–Raphson solving technique. To the best of the authors’ knowledge, no paper exists that has studied large deflections of a piezomagnetic nanosize beam with apparent flexomagneticity, unless otherwise stated. Estimations hereon take the necessary properties for a piezomagnetic nanoparticle accorded by Table 3 as [13,14].

In light of the lack of sufficient study on FM, we took *f*_31_ = 10^−9^ N/Ampere, *f*_31_ = 10^−10^ N/Ampere as [13,14]. These two values were also theoretically obtained based on some simple assumptions and cannot be the exact numeric values of the flexomagnetic parameter of the aforesaid material presented in Table 3.

An NSGT case was chosen to consider nanoscale impacts. In this model, as can be observed by Equation (31), there were two small scale factors. In point of fact, to determine the results of the bending of the nanoparticle, the amounts of these two parameters are vital. Thus, by exploring within the literature, one can find the 0.5 nm < *e_0_a* < 0.8 nm [46], and 0 < *e_0_a* ≤ 2 nm [47,48], unless otherwise stated. The amount of strain gradient parameter was obtained in a similar size to the lattice parameter of the crystalline structure [49]. This factor for the aforementioned material in Table 3 was obtained in an experiment to change between 0.8 and 0.9 nanometers at a set temperature [50]. Hence, the averaged value of the strain gradient parameter is selected as *l* = 1 nm.

#### 4.2.1. Effect of Nonlinearity

**Figure 2:**
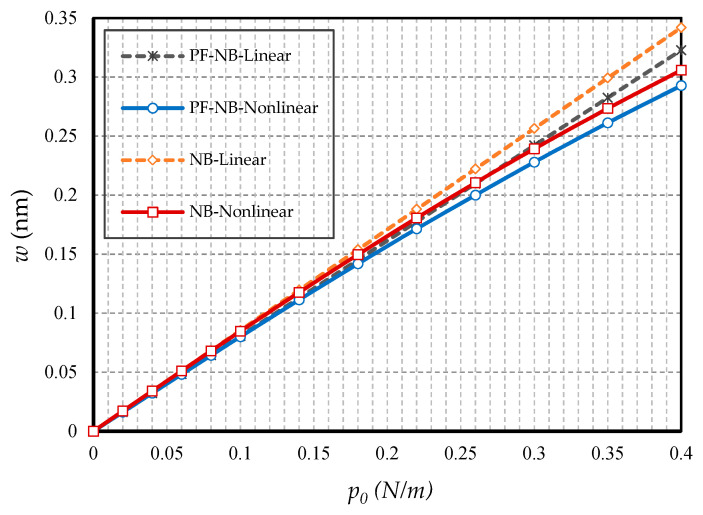
Transverse load vs. different cases of nanobeams (*Ψ* = 1 mA, *l* = 1 nm, *e_0_a* = 0.5 nm, C-C).

**Figure 3:**
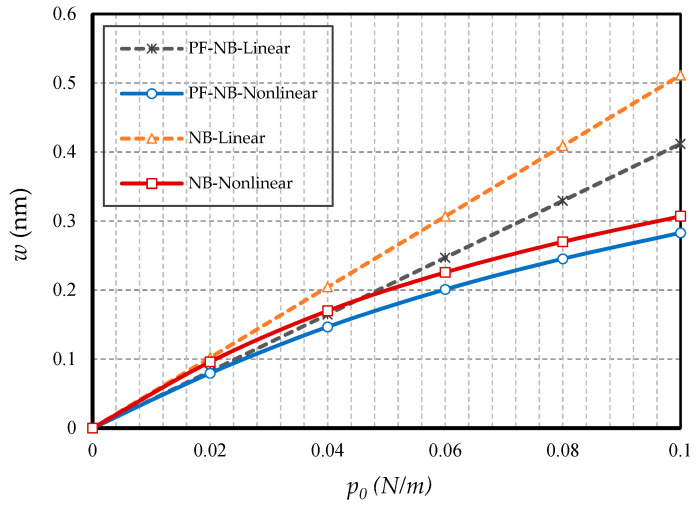
Transverse load vs. different cases of nanobeams (*Ψ* = 1 mA, *l* = 1 nm, *e_0_a* = 0.5 nm, S-S).

**Figure 4:**
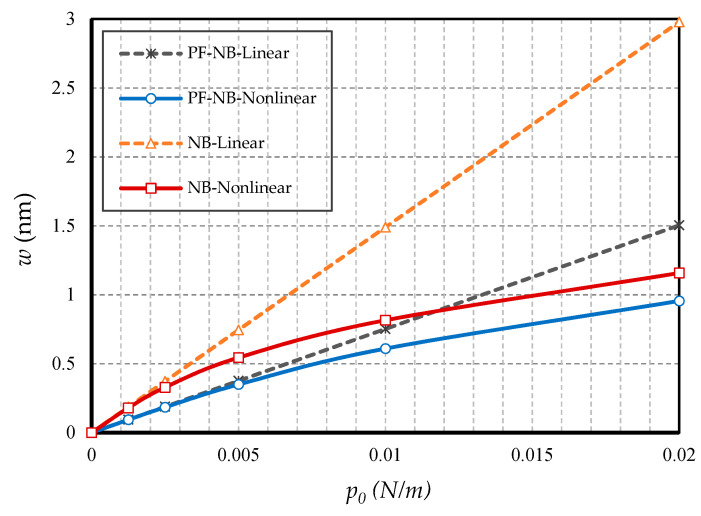
Transverse load vs. different cases of nanobeams (*Ψ* = 1 mA, *l* = 1 nm, *e_0_a* = 0.5 nm, C-F).

To probe the numerical results, we first show the difference between the results of the linear and nonlinear analyses. Figure 2 is provided for the fixed support, Figure 3 is produced for the hinge support, and lastly, Figure 4 is presented for the cantilever nanobeam. It should be noted that all figures in the results section were plotted in both linear and nonlinear modes for the piezomagnetic nanobeam (P-NB), piezomagnetic-flexomagnetic nanobeam (PF-NB), and common nanobeam (NB). Let us come back to Figure 2, Figure 3 and Figure 4. First, a comparison of the figures shows a much smaller deflection which resulted from the boundary condition of the fix versus the other ones. For this reason, a larger load amplitude was selected to evaluate the results of the fixed–fixed support to better distinguish between linear and nonlinear analyses. In the first figure, as can be seen, the results of the linear analysis were valid as long as the deflection value did not reach 15% of the thickness, i.e., *w* < 0.15 *h*. Of course, it is important to note that according to the second figure and in the boundary condition of the hinge, this value was *w* ≤ 0.1 *h* for NB and *w* ≤ 0.08 *h* for PF-NB. This means that if the deflections exceed these values, the linear analysis is no longer valid, and we must use nonlinear analysis to examine the nanobeam’s deflections. Considering Figure 4 for a more flexible beam with clamped-free end conditions represents that the allowable value for NB was about *w* ≤ 0.2 *h* and for PF-NB, about *w* ≤ 0.1 *h*. It is relevant to state that due to the C-F case, a very small lateral load was chosen because of the high deflection capacity of the nanobeam in free conditions. Comparing the three figures, it is interesting to note that the difference between the results of the linear and nonlinear analyses was greater in, respectively, C-F > S-S > C-C boundary conditions, and the C-F boundary condition was more sensitive. It may be concluded that nanobeams with end conditions with higher degrees of freedom require a more urgent nonlinear analysis. Another result of these diagrams is that the deflections of magnetic nanobeam in both linear and nonlinear analyses were smaller than that of the conventional nanobeam. In addition, the difference between the results of the linear analysis was greater than that of the nonlinear analysis. These results strongly suggest that nonlinear strains must be used for static deflection analysis in materials, unless the loads are selected so that the deflections are within the range obtained for linear analysis. By carefully examining the results in [14], which is based on linear analysis and a thickness of 10 nm, it can be seen that the deflections in some diagrams of this reference (see Figure 3 of the reference) were within the range, and in some others exceeded the obtained range (see Figure 4 of the reference). Therefore, the linear analysis cannot always be valid, and certainly, nonlinear analysis is a matter of need.

#### 4.2.2. Effect of Small Scale

**Figure 5:**
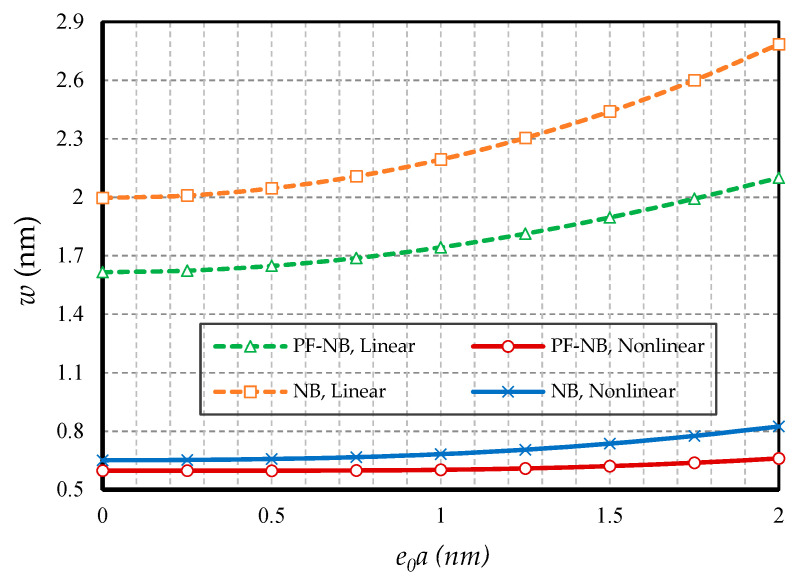
Nonlocal parameter vs. different cases of nanobeams (*Ψ* = 1 mA, *l* = 1 nm, *p_0_ =* 0.4 N/m, S-S).

**Figure 6:**
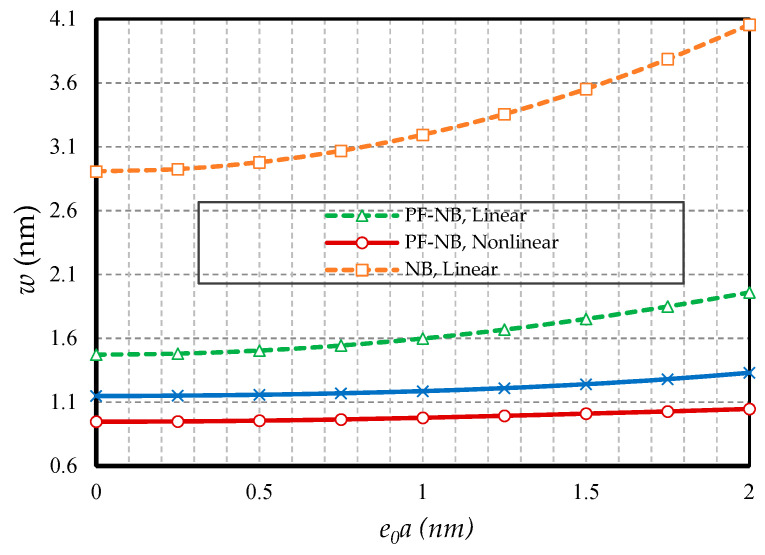
Nonlocal parameter vs. different cases of nanobeams (*Ψ* = 1 mA, *l* = 1 nm, *p_0_ =* 0.02 N/m, C-F).

**Figure 7:**
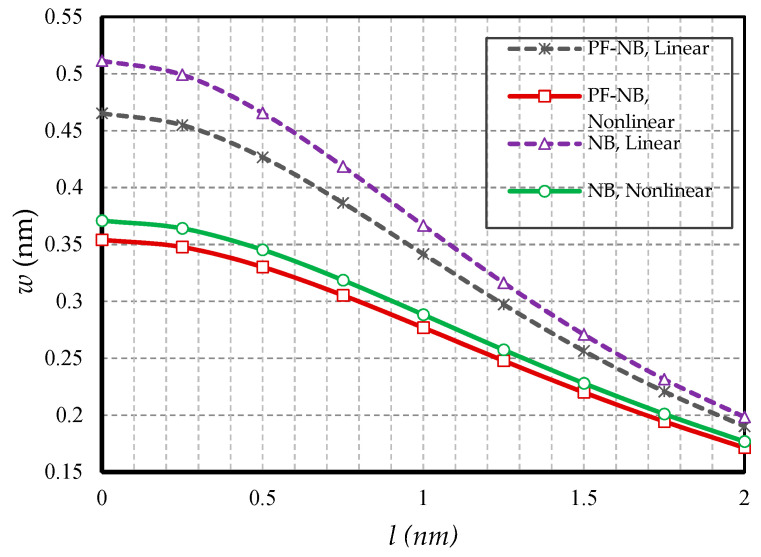
Strain gradient parameter vs. different cases of nanobeams (*Ψ* = 1 mA, *e_0_a* = 1 nm, *p_0_ =* 0.4 N/m, C-C).

**Figure 8:**
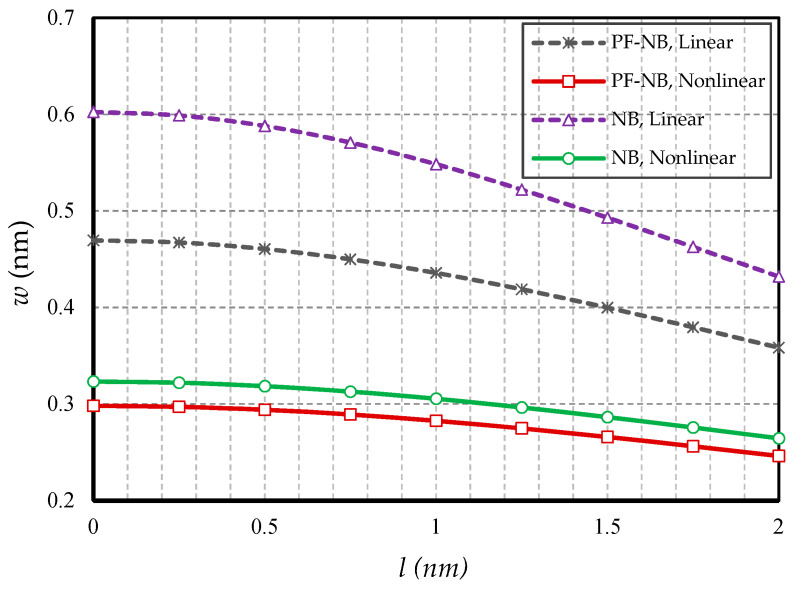
Strain gradient parameter vs. different cases of nanobeams (*Ψ* = 1 mA, *e_0_a* = 1 nm, *p_0_ =* 0.1 N/m, S-S).

In this section, the effect of small-scale parameters is examined, i.e., nonlocal and strain gradient parameters. Figure 5 and Figure 6 show the effect of variations in the value of the nonlocal parameter, respectively, for S-S and C-F, and Figure 7 and Figure 8 exhibit the effect of changes in the value of the strain gradient parameter, respectively, for C-C and S-S. The first and second figure show that as the nonlocal parameters increased, the deflections increased in all four cases examined. As a result, it can be stated that the increase in the nonlocal parameter had a softening effect on the nanobeam material. On the other hand, it is worth noting that as the numerical value of the nonlocal parameter increased, this caused the difference between the linear and nonlinear analyses results. In fact, in the nonlocal analysis of nanobeams, the effect of nonlinear analysis will be greater, and this requires that nonlinear analysis be used to investigate nonlocal deflections. It is important to note that the effect of the nonlocal parameter on the results of magnetic nanobeam was greater than that of the conventional nanobeam. This result is due to the steeper slope of the results of this nanobeam with the increasing nonlocal parameter. It is also interesting to say that the difference between the results of nonlinear and linear analyses in NB was much more than in PF-NB. From the third and fourth figures, which show the effect of changes in the strain gradient parameter in two different boundary conditions, it is clear that increasing this parameter led to a decrease in deflections of all cases and means that the increase in the strain gradient parameter is a tightening effect inside the material. However, it is important to bear in mind that this tightening effect will be greater in the case of a boundary with lower degrees of freedom. As can be observed, in a nanobeam with a double-sided fixed boundary condition, the slope of the reduction in the deflection’s results was much faster than in the case of the boundary conditions of the double-sided hinged. It is also interesting to note that increasing the numerical value of the strain gradient parameter will reduce the difference between the results of linear and nonlinear analyses, and in very large values of this parameter, it can be explicitly stated that nonlinear analysis can be ignored provided that small loads are applied.

#### 4.2.3. Effect of Magnetic Field

**Figure 9:**
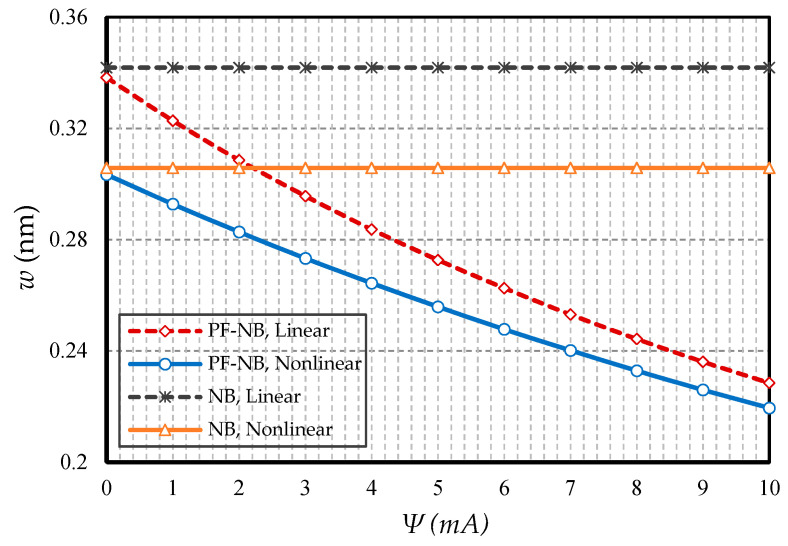
Magnetic potential parameter vs. different cases of nanobeams (*l* = 1 nm, *e_0_a* = 0.5 nm, *p_0_ =* 0.4 N/m, C-C).

**Figure 10:**
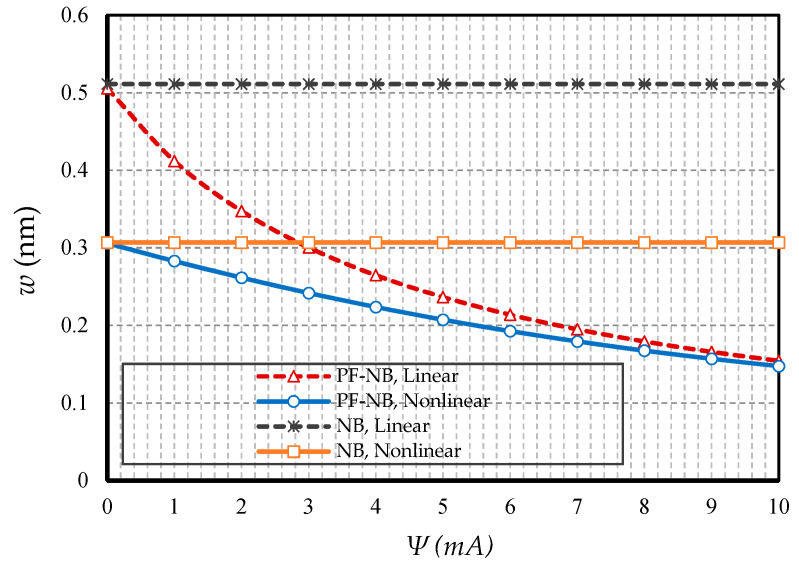
Magnetic potential parameter vs. different cases of nanobeams (*l* = 1 nm, *e_0_a* = 0.5 nm, *p_0_ =* 0.1 N/m, S-S).

**Figure 11:**
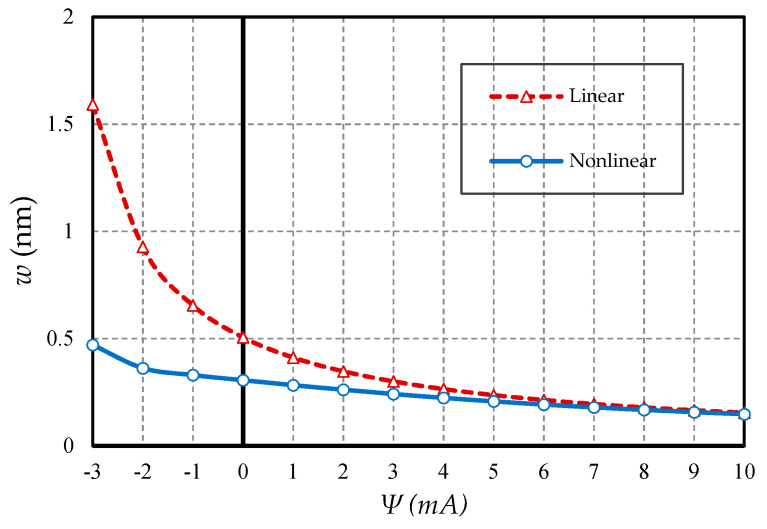
Magnetic potential parameter vs. PF nanobeams (*l* = 1 nm, *e_0_a* = 0.5 nm, *p_0_ =* 0.1 N/m, S-S).

The effect of the external magnetic field was dominant in the mechanical analysis of materials with flexomagnetic capability, while the magnetic effect was inverse. For this purpose, based on Figure 9 and Figure 10, the effect of increasing the magnetic potential in the positive magnetic field is presented in two boundary condition states. Naturally, since the ordinary nanobeam does not have piezomagnetic properties, increasing the magnetic potential will have no effect on this material model. For this reason, the deflections of NB in different values of the external magnetic potential are constant. However, in piezo-flexo nanobeams, with increasing external magnetic potential, the deflections decreased in both linear and nonlinear states in both boundary conditions. Perhaps it can be interpreted that the effect of the magnetic field shrinks the material, and eventually, the material became stiffer and in the case of contraction, most of the deflections became smaller. As can be seen, in the linear analysis case, the difference in results of the conventional and magnetic nanobeams was more visible. In fact, linear analysis showed external effects with a slight exaggeration. Another interesting point is that increasing the potential of external magnetic led to convergence of the results of linear and nonlinear analyses in the piezo-flexomagnetic nanobeam, but this convergence occurred faster in the boundary condition of the hinge, so much so that in small amounts of external magnetic potential, the results of the linear and nonlinear analyses were perfectly matched to each other. Figure 11 is also displayed to show the impact of a negative magnetic field. The general conclusion that can be drawn from these three figures is that in a positive magnetic field the effect of nonlinear analysis decreases and in contrast in a negative magnetic field the influence of nonlinear analysis will be very prominent.

#### 4.2.4. Effect of Slenderness Ratio

**Figure 12:**
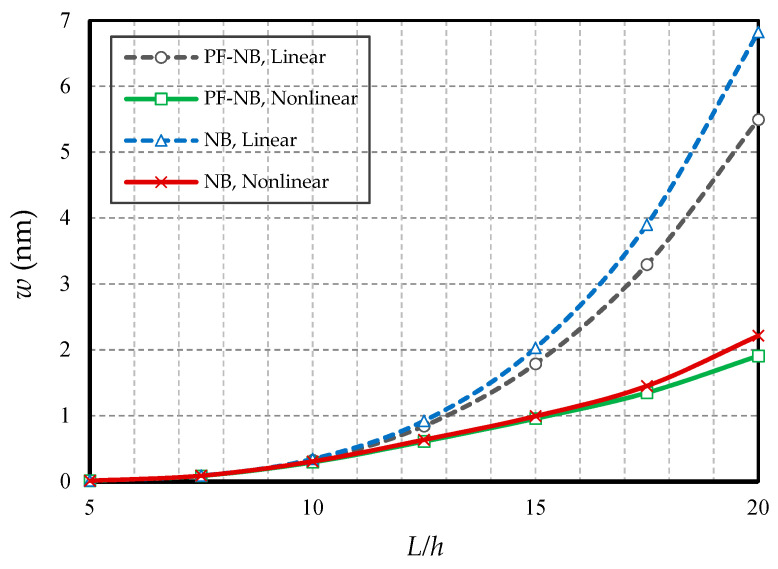
Slenderness ratio vs. different cases of nanobeams (*Ψ* = 1 mA, *l* = 1 nm, *e_0_a* = 0.5 nm, *p_0_ =* 0.4 N/m, C-C).

**Figure 13:**
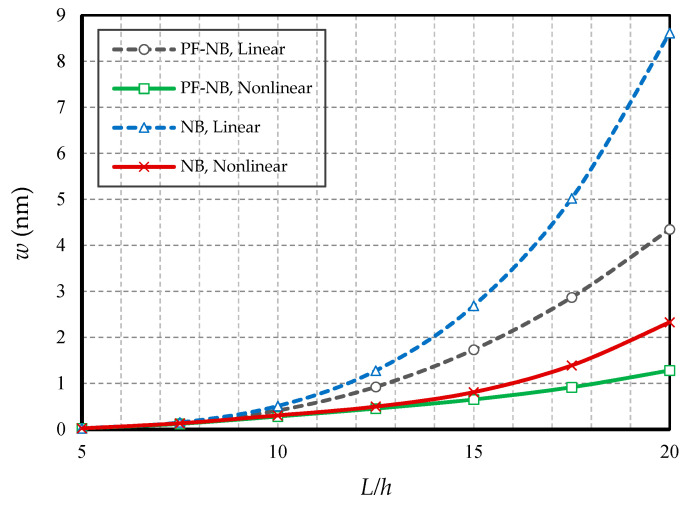
Slenderness ratio vs. different cases of nanobeams (*Ψ* = 1 mA, *l* = 1 nm, *e_0_a* = 0.5 nm, *p_0_ =* 0.1 N/m, S-S).

Figure 12 and Figure 13 are drawn by defining the ratio of length to thickness as a slenderness coefficient in the nanobeam. The first figure is reported for the boundary condition of the two heads of fix and the second figure is plotted for the two heads of the hinge. As can be easily seen, increasing the slenderness ratio led to an increase in static deflections in both linear and nonlinear states. Additionally, with increasing this coefficient of the nanobeam, the difference between the results of linear and nonlinear analyses increased significantly. In fact, this suggests that in large quantities of length, the linear analysis presented completely erroneous results. On the other hand, in large quantities of slenderness coefficient, the difference between the results of the magnetic nanobeam and common nanobeam in linear mode were greater than in the nonlinear one, which proves that in large values of length, the linear results showed, with magnification, the mechanical behavior of the magnetic nanobeam versus the conventional nanobeam, and it cannot be true. It should be emphasized that this difference was much greater in the results of the hinge boundary condition even with smaller loads, than in the results of the clamp boundary condition.

#### 4.2.5. Effect of FM

**Figure 14:**
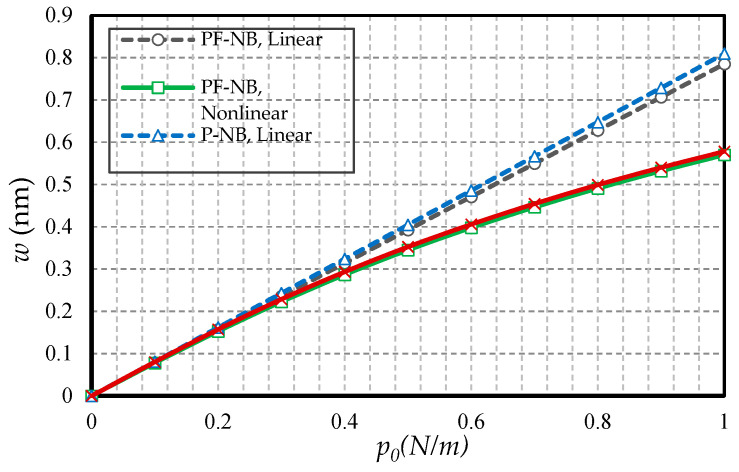
Transverse load vs. deflection for different cases of nanobeams (*Ψ* = 1 mA, *l* = 1 nm, *e_0_a* = 0.5 nm, C-C).

**Figure 15:**
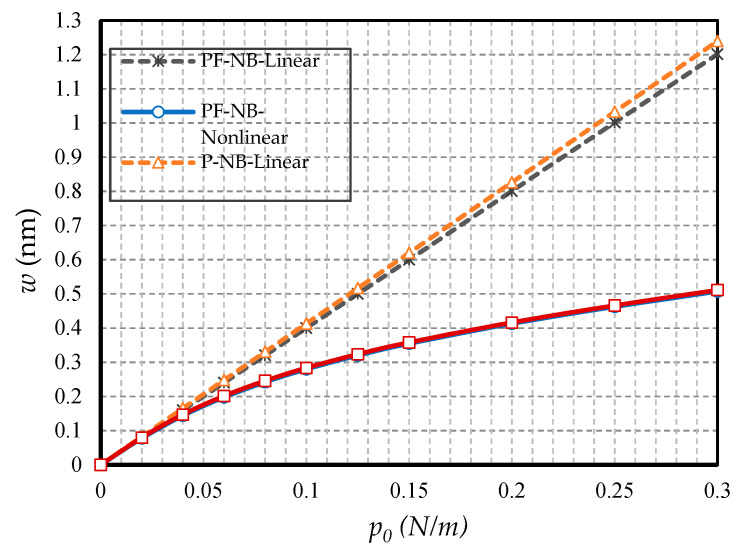
Transverse load vs. deflection for different cases of nanobeams (*Ψ* = 1 mA, *l* = 1 nm, *e_0_a* = 0.5 nm, S-S).

**Figure 16:**
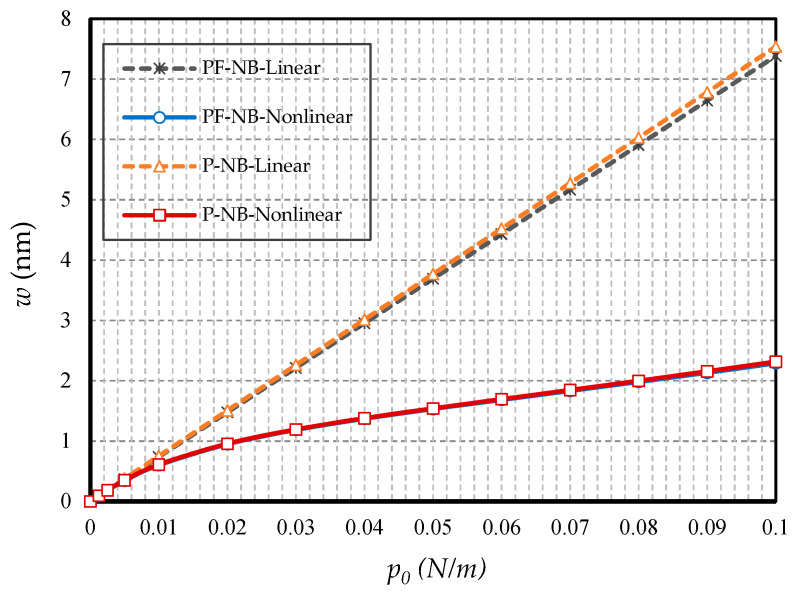
Transverse load vs. deflection for different cases of nanobeams (*Ψ* = 1 mA, *l* = 1 nm, *e_0_a* = 0.5 nm, C-F).

In this subsection, the aim is to compare the difference in results when the substance has only a piezomagnetic effect when the flexomagnetic effect is added to it. Figure 14 shows the results of the nanobeam with two side clamps; in Figure 15, the nanobeam with two ends of the hinge is presented; finally, Figure 16 shows the cantilever nanobeam. First, as can be seen, the nonlinear analysis reduced the flexomagnetic effect. This result was obtained from the difference between the results of the P-NB and PF-NB in both nonlinear and linear analyses of the figures. On the other hand, as is clear, the results associated with the PF-NB were smaller than those of the P-NB. This finding can be interpreted in such a way that the flexomagnetic effect will lead to more material stiffness, and as a result, the deflections will be smaller while considering this effect. It has to be noted that the slight difference in the results of P-NB versus those of the PF-NB was directly related to the values of the flexomagnetic modulus. According to the references, the value of the parameter was almost based on the assumptions, and due to the novelty, of the discovery of the flexomagnetic effect; the exact values of this parameter have not yet been calculated. For this reason, it is not possible to say why the difference in results between P-NB and PF-NB was high or low. Nevertheless, such a difference was also adequately large on a nanoscale. It should be pointed out that the FM was more remarkable in C-C end conditions. This means that the lower degree of freedom boundary condition increased the impact of FM.

**Figure 17:**
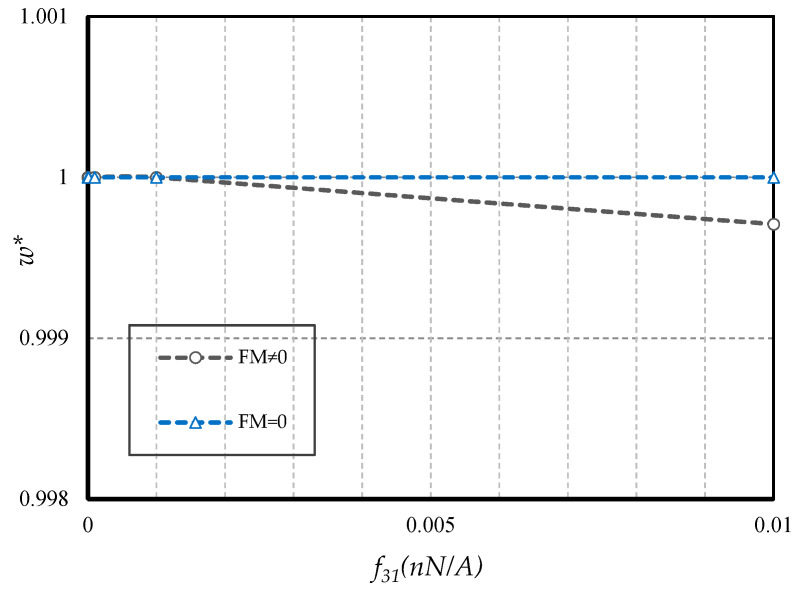
Presence and absence of flexomagnetic modulus for linear bending of a PF-NB (*Ψ* = 1 mA, *l* = 1 nm, *e_0_a* = 0.5 nm, *p_0_ =* 0.5 N/m, S-S).

In this study, we end the discussion with Figure 17, in which different values of the flexomagnetic parameter were investigated. To carry out this, the *w* * was introduced which was the deflections of the PF-NB divided by the deflections of the P-NB. As seen, there was no appreciable change in deflections originated from FM in lower amounts of the FM parameter. The effect of FM on the P-NB became outstanding for large values of FM, and the assumed value *f*_31_ = 10^−10^ N/Ampere can affect to some extent the behavior of the PF-NB.

## 5. Conclusions

Due to the FM influence being new and interesting, we took into account both piezomagnetic and flexomagnetic effects together for a reduced scale thin beam. The geometrical nonlinearity which induces the large deformations was also assessed. Applying the variational formulation derived the favourable governing equations. To capture the consistent nanoscale effect, the NSGT was inserted into the mathematical model. Transmuting the acquired relations based on the NSGT into the displacement relationship gives an eligible equation, which stands to compute large deflections. The translation and shifting of the nonlinear system of ordinary differential equations into the algebraic ones were performed based on the GRWM. The GRWM concerning an analytical flow estimated clamped, simply-supported, and free end conditions. Afterward, the numerical solution regarding NRT was investigated. From the obtained results, one can briefly write

- In hinged–hinged nanobeams, linear deflections for a NB can be used in the range *w* ≤ 0.1 *h*, and for a PF-NB, about *w* ≤ 0.08 *h*. This value in a double-fixed NB and PF-NB is in the range *w* < 0.15 *h*. However, for a cantilever case in NB, it is *w* ≤ 0.2 *h* and in PF-NB, it is *w* ≤ 0.1 *h*.
- The difference between the nonlinear analysis and the linear one will be more pronounced in the boundary condition with higher degrees of freedom.
- Increasing the numerical value of the nonlocal parameter leads to a softening effect on the material, and in contrast, increasing the numerical value of the strain gradient parameter leads to the appearance of stiffness in the material.
- The effect of nonlinear analysis is greater in large values of nonlocal parameters and small values of strain gradient parameters.
- The effect of nonlinear analysis on a nonlocal study is greater than a local one.
- The effect of nonlinear analysis in the positive magnetic field decreases. However, the opposite is true in the case of a negative magnetic field.
- For nanobeams with very large lengths, linear analysis gives entirely erroneous results even if the values of lateral loads are not large.
- The flexomagnetic effect leads to more material stiffness, and thus reduces the numerical values of deflections in static analysis.
- The less flexible the boundary condition, the higher the flexomagneticity effect.

## Figures and Tables

**Table 1:** Dimensionless maximum deflection for a simply-supported nanobeam exposed to transverse uniform loading.

*L*/*h*	*e*_0_*a*/*L*	EBT, Linear [21]	EBT, Linear [45]	EBT, Linear [Present]
10	0	0.013021	0.013021	0.013021
	0.05	0.013333	0.013333	0.013333
	0.1	0.014271	0.014271	0.014271
	0.15	0.015833	0.015833	0.015833

**Table 2:** Maximum deflection (mm) for a clamped–clamped macro beam exposed to transverse uniform loading (E=210 GPa, h=5 mm).

*L*/*h*	*p* (kN/mm)	EBT, Linear [Present]	FEM, Linear [ABAQUS]
10	0.01	0.0792	0.0824
	0.02	0.1585	0.1648
	0.03	0.2377	0.2472
	0.04	0.3170	0.3297

**Table 3:** Engineering necessary features of a piezomagnetic nanobeam with apparent flexomagneticity.

CoFe_2_O_4_
*C*_11_ = 286 GPa
*q*_31_ = 580.3 N/Ampere.m
*a*_33_ = 1.57 × 10^−4^ N/Ampere^2^
*L* = 10 *h*
